# Machine learning algorithms in the estimation of sex from 3DCT-generated cranial and pelvic measurements

**DOI:** 10.1007/s00414-026-03890-2

**Published:** 2026-07-02

**Authors:** Oladiran I. Olateju, Oriasotie M. Ujaddughe, Muhammad E. H. Chowdhury, Mubarak A. Bidmos

**Affiliations:** 1 Department of Anatomical Sciences, School of Biomedical Sciences, Faculty of Health Sciences, University of the Witwatersrand, Johannesburg, South Africa; 2 Department of Electrical Engineering, College of Engineering, Qatar University, Doha, Qatar; 3 Department of Basic Medical Sciences, College of Medicine, QU Health, Qatar University, Doha, Qatar

**Keywords:** Forensic anthropology, Sex estimation, Cranium, Pelvic bone, Machine learning algorithms

## Abstract

Sex estimation from skeletal remains is a key component of forensic anthropology, with the skull and pelvis being the most sexually dimorphic elements in terms of morphology. Traditional morphometric approaches, such as discriminant function and logistic regression analyses, have achieved high accuracy in estimating sex across various populations, including South Africans. However, the use of machine learning (ML) for sex estimation based on cranial and pelvic measurements has not yet been explored in any South African population. This study assessed the potential of ML algorithms to estimate sex from cranial and pelvic measurements derived from computed tomography (CT) scans of contemporary Black South Africans. The sample included 680 skeletal elements (400 crania and 280 pelvic bones) with an equal distribution of males and females. CT scans archived at the Department of Radiology, Charlotte Maxeke Johannesburg Academic Hospital, were reconstructed into 3D models using Xiris and IntelliSpace software, from which eight cranial and eleven pelvic measurements were collected. Seven classical ML algorithms were applied and feature ranking techniques were used to determine the most informative variables for sex estimation. A stacking ML model was then developed, incorporating the top three classifiers as base learners. Their outputs were combined and passed to different ML algorithms acting as meta-learners for final classification. The findings confirmed strong sexual dimorphism in cranial and pelvic bones, and the stacking models achieved superior accuracy (cranium: 80.3–94.3%; pelvis: 86.1–96.1%) compared to traditional multivariate methods, demonstrating the potential of ML in forensic sex estimation.

## Introduction

Sex estimation from recovered skeletal remains is a vital first step in the human identification process carried out by forensic anthropologists. This estimation plays a crucial role in narrowing the pool of potential identities when constructing the biological profile of unidentified remains [1]. Alongside age, stature, and population affinity, sex estimation is fundamental to establishing an individual’s identity from skeletal material [1]. Of all skeletal elements, the skull and pelvis exhibit the most pronounced morphological differences between sexes across diverse populations, and these traits have consistently demonstrated high accuracy in sex estimation [2]. Both early research [3, 4] and more recent studies [5] have validated the reliability of these bones in distinguishing biological sex. However, traditional morphological assessment is largely subjective and relies heavily on the examiner’s experience, often requiring many years of practice. As a result, there has been a growing shift toward quantifying these differences through objective approaches, such as geometric morphometrics [6–8].

Beyond morphological assessment, standard osteometric measurements can be analyzed using classical statistical techniques like logistic regression and discriminant function analysis. These statistical models are known to be population-specific, meaning they yield the most reliable results when applied to the same population from which they were developed. Consequently, various population-specific standards have been established using skeletal measurements from different bones, including the skull [9–12], vertebral column [13–15], pelvis [16–18], upper limb long bones [19–22], lower limb long bones [23–26], and bones of the hand and foot [27–29]. In South Africa, similar research efforts have produced standards tailored to local populations for sex estimation using measurements of the skull [30, 31], long bones of the limbs [32–36], pelvis [37], and foot bones [38, 39]. These standards were primarily derived from skeletal remains of Black South Africans and White South Africans housed in collections such as the Raymond A. Dart Collection of Human Skeletons [40], the Pretoria Bone Collection [41], and the University of Cape Town (UCT) Osteological Collection [42].

In the past decade, machine learning (ML) has become increasingly prominent in developing clinical sex estimation models, particularly for forecasting health outcomes based on medical data [43–45]. Recent literature reviews have highlighted the growing interest in applying ML to clinical decision-making and underscored the ongoing need for robust prognostic research [46]. ML offers several advantages, including the ability to efficiently process large and complex datasets and uncover subtle patterns not easily detected by clinicians [47]. Furthermore, while many ML approaches are flexible and nonparametric, certain commonly used methods, such as discriminant function analysis and logistic regression, are parametric and rely on underlying assumptions about data distributions [48, 49] Nonetheless, ML techniques can accommodate numerous input variables simultaneously and remain widely valued for their adaptability in modelling complex relationships [48, 49].

Machine learning methods are increasingly being applied to estimate biological profiles from skeletal remains. Recent models trained on cranial [50–52] and pelvic [53–55] measurements show high classification accuracy and often outperform traditional sex‑estimation techniques. However, in South Africa, discriminant function analysis is still commonly used, and the use of advanced ML methods remains limited. Population‑specific models are essential for forensic accuracy, and Black South Africans, who make up the country’s largest population group and remain the most socio‑economically disadvantaged, experience persistent economic inequalities that influence both skeletal variation and medico‑legal identification outcomes [56]. They also represent the largest absolute number of crime‑affected individuals because they are disproportionately concentrated in high‑crime, high‑inequality urban precincts, particularly metropolitan municipalities where most recorded crimes occur [56, 57]. Violent crime is further strongly linked to intra‑group inequality within Black African communities, highlighting their heightened exposure to risk [56]. Against this background, the present study develops ML‑based sex‑estimation models using CT‑derived cranial and pelvic measurements from contemporary Black South Africans and evaluates their performance against classical statistical techniques, addressing a critical forensic and public‑health need for robust, population‑appropriate methods.

## Materials and methods

An ethical clearance waiver (M220108) was granted by the Human Research Ethics Committee (HREC) (Medical) at the University of the Witwatersrand, Johannesburg, South Africa. This waiver permitted the use of pre‑existing data that had already been collected and analysed for the development of discriminant function and logistic regression models for sex estimation based on cranial [58] and pelvic [59] measurements. In granting the waiver, the Committee adhered to national and international ethical frameworks, including the principles outlined in the Declaration of Helsinki, which underpin the institution’s Good Clinical Practice requirements. The dataset consisted of complete CT scan records from 680 Black South African individuals aged 18 to 60 years. These records, obtained between January 2016 and December 2022 and archived in the Radiology Department of the Charlotte Maxeke Johannesburg Academic Hospital (CMJAH), Johannesburg, South Africa, were retrospectively evaluated for this study [58, 59]. As the dataset was derived from CT scans of patients who had visited the hospital for clinical purposes, a Consent to Participate declaration was not required. Individuals older than 60 years were excluded due to their increased susceptibility to osteoporosis and age‑related reductions in bone density, which could potentially affect skeletal measurements [60]. The final sample consisted of 400 head CT scans (200 males, 200 females) [58] and 280 pelvic CT scans (140 males, 140 females) [59]. All CT scans were acquired using a SOMATOM Definition AS CT scanner (64-slice; 120 kVp; 100 mA) with a slice thickness of 1.0 mm and were stored in Digital Imaging and Communications in Medicine (DICOM) format. Image acquisition and processing were performed using Xiris and IntelliSpace PACS Radiology software (version 4.4.516.21; Philips Healthcare, Best, Netherlands) [58, 59].

Table 1 presents the distribution of cranial and pelvic bone samples by sex and age group. Among male cranial samples, the highest frequencies (*n* = 29) were found in the 26–30 and 41–45 age groups, whereas the female cranial samples were most represented in the 56–60 age group (*n* = 39). For the pelvic bone dataset, the 26–30 age group had the most male samples (*n* = 30), while the 56–60 age group also had the highest number of female samples (*n* = 31), reflecting a similar distribution to the cranial data. The 16–20 age group had the fewest samples for both sexes across both datasets. Overall, the cranial and pelvic bone datasets maintained a balanced distribution of male and female samples across the various age categories. The definitions of the cranial and pelvic bone measurements are presented in Table 2.

**Table 1:** Description of samples based on age group and sex[58, 59

Age group (years)	Cranium		Pelvic bone	
	M (*n* = 200)	F (*n* = 200)	M (*n* = 140)	F (*n* = 140)
16–20	5	11	1	4
21–25	20	14	14	14
26–30	29	25	30	11
31–35	35	26	16	13
36–40	28	32	29	19
41–45	29	14	18	18
46–50	18	19	7	15
51–55	12	20	13	15
56–60	24	39	12	31
Mean ± SD	34.0	31.7	38.0	42.7
	13.7	7.1	11.0	12.5

**Table 2:** Definition of cranial and pelvic bone measurements [58, 59

S/*N*		Abbreviation	Description
	Cranial measurement[58]		
1	Maximum cranial length	CRA_01	The linear distance between the glabella and the opisthocranion is taken in the midsagittal plane.
2	Basi-bregmatic height	CRA_05	The linear distance between the basion and the bregma is taken in the midsagittal plane.
3	Basion–nasion length	CRA_06	The linear distance between the basion and the nasion.
4	Bi-zygomatic breadth	CRA_04	The widest linear distance on the arch of the two zygomatic bones.
5	Nasal height	CRA_14	The linear distance between the nasion and the nasospinale is taken on the midline.
6	Nasal breadth	CRA_15	The maximum breadth of the nasal aperture – Alare to Alare.
7	Maximum frontal breadth	CRA_12	The linear distance between the two coronal sutures.
8	Nasion-Prosthion Length	CRA_11	The linear distance between the nasion and the prosthion is taken on the midline.
	Pelvic bone measurement [59]		
1	Pubic length	PuL	Measured from the highest point on the superior ridge of the acetabulum representative of the centre of origin of the iliac blade to the most superior and medial point on the pubic crest.
2	Ischial length	IsL	Measured from the highest point on the superior ridge of the acetabulum representative of the centre of origin of the iliac blade to the deepest point on the ischial tuberosity.
3	Total height	THox	The greatest distance from the most superior point on the iliac crest to the most inferior point of the ischial tuberosity
4	Iliac breadth	IlB	The greatest distance from the anterior superior to the posterior superior iliac spines.
5	Greater sciatic notch breadth	GSNb	Measured from the base of the ischial spine to the posterior inferior iliac spine stopping at a point before the curvature of the spine angles to the posterior.
6	Greater sciatic notch depth	GSNd	This is taken as the distance between the GSNb and the greatest depth of the notch in such a way that gives the maximum depth perpendicular to the GSNb.
7	Acetabular diameter	AceDia	Maximum diameter of the acetabulum measured in a superior-inferior direction.
8	Obturator foramen height	OFH	Measured from the most inferior point within the foramen to the most superior point at the superior pubic ramus.
9	Obturator foramen width	OFW	Measured perpendicular to the height from the posterior to the anterior borders of the foramen.
10	Pubic height	PuH	Measured from the most superior to the most inferior point on pubic symphysis.
11	Pubic width	PuW	Measured on the dorsal aspect of the bone from the most inferior point on the face of the pubic symphysis, horizontally to the medial aspect of the obturator foramen.

Inter-observer reliability was assessed using repeated measurements on randomly selected cranial and pelvic CT scans, with the calculated relative technical error of measurement (rTEM) for both datasets summarised in Table 3. Acceptable levels of measurement error were observed across all measurements when considered in relation to measurement size and allowable margins of error, indicating a high degree of measurement reliability [61, 62]. According to Bruton et al. [61] and Weinberg et al. [62], measurement reliability is best evaluated using rTEM, with values below 2–2.5% considered acceptable for inter-observer comparisons.

**Table 3:** Inter-observer Technical Error of Measurement (rTEM) of the measurements of the cranial [58] and pelvic bone [59]

Cranial measurements[58]	rTEM
CRA_01 (Maximum Cranial Length)	0.270
CRA_05 (Basi-Bregmatic Height)	0.530
CRA_06 (Nasion-Basion L. aka Cranial Base L)	0.680
CRA_04 (Bi-Zygomatic Breadth)	1.250
CRA_14 (Nasal Height)	1.110
CRA_15 (Nasal Breath)	2.060
CRA_12 (Maximum Frontal Breath)	0.860
CRA_11 (Nasion– prosthion length)	1.530
Pelvic bone measurements [59]	rTEM
PuL (Pubic length)	0.056
IsL (Ischial length	0.030
THox (Total height)	0.007
IlB (Iliac breadth)	0.010
GSNb (Greater sciatic notch breadth)	0.022
GSNd (Greater sciatic notch depth)	0.029
AceDia (Acetabular diameter)	0.024
OFH (Obturator foramen height)	0.053
OFW (Obturator foramen width)	0.007
PuH (Pubic height)	0.027
PuW (Pubic width)	0.017

### Statistical analysis

Statistical analyses were performed using Stata/MP version 13.0 and SPSS version 23. Descriptive statistics were computed for each cranial and pelvic bone measurement, categorized by sex. These included the sample count (n), mean (M), standard deviation (SD), median (MDN), minimum (MIN), maximum (MAX), and interquartile range (Q1 and Q3), with the results summarized in Table 4 for cranial measurements and Table 5 for pelvic measurements. A significance threshold of *p* < 0.05 was applied throughout.

**Fig. 1:**
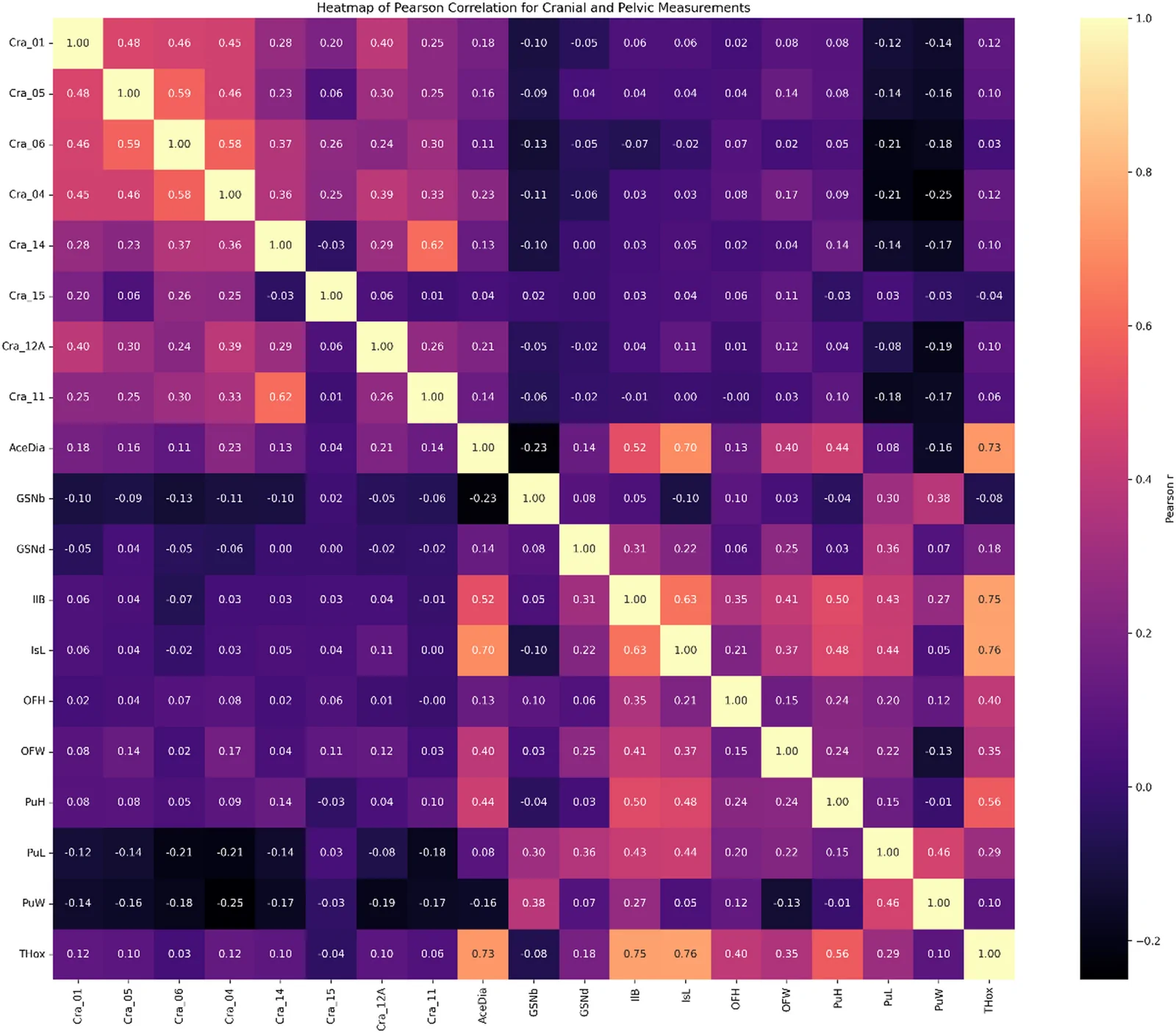
Heatmap of Pearson correlation among different features

The analysis covered eight cranial and eleven pelvic variables. Pearson correlation coefficients were calculated to assess the strength of linear relationships between variables. Correlation magnitudes were interpreted according to the classification proposed by Chan [63], where coefficients of 0.00–0.19 indicate very weak, 0.20–0.39 weak, 0.40–0.59 moderate, 0.60–0.79 strong, and 0.80–1.00 very strong relationships. As illustrated in the correlation heatmap (Fig. 1), most measurement pairs demonstrated weak-to-moderate associations. The highest observed correlation was between IsL and THox (*r* = 0.76), representing a strong positive relationship, followed by IlB and THox (*r* = 0.75) and IsL and IlB (*r* = 0.63), which also indicated strong associations. Among cranial variables, the strongest correlation was observed between Cra_04 and Cra_05 (*r* = 0.58), representing a moderate relationship.

Variables exhibiting low-to-moderate correlations were retained because they likely contribute complementary information rather than redundant information to the predictive models. Retaining such variables allows machine learning algorithms to exploit unique patterns across measurements and potentially improve classification performance while minimizing multicollinearity-related issues [64]. Recent studies [52, 54, 65] have shown that many machine learning algorithms, particularly ensemble-based approaches such as Random Forest, Extra Trees, and gradient boosting models, are relatively robust to moderate multicollinearity and can benefit from preserving complementary predictors that capture distinct information content and complex feature interactions. Additionally, maintaining informative variables can improve model generalization and predictive stability, particularly in biomedical and forensic applications involving multidimensional datasets.^66^ This strategy is particularly relevant for ensemble learning models, which can effectively leverage weakly correlated variables to capture non-linear interactions and improve discrimination performance [66].

## Data normalization

The performance of machine learning models is heavily influenced by the quality and uniformity of the input data. A key pre-processing step is normalization, which ensures that all features contribute equally during training which is a factor known to enhance model accuracy [45]. In this study, Z-score normalization was applied, as it is effective in minimizing the impact of outliers. The formula used is shown in Eq. 1.

1 $$\:v'\:=\frac{v-{\mu\:}_v}{{\sigma\:}_v}$$

where v’ is the standardized value, v is the original value, and µ_v and σ_v are the mean and standard deviation of the variable within the training data. This method ensures the data is centred around a mean of zero with a standard deviation of one.

## Top-ranked feature identification

Feature selection is used to identify the most relevant variables for sex estimation, helping to reduce overfitting, improve model accuracy, and speed up training. Several feature-selection methods are widely used in machine learning applications, including univariate selection, Recursive Feature Elimination (RFE), Principal Component Analysis (PCA), and ensemble-based methods. These approaches have demonstrated effectiveness in reducing dimensionality and improving predictive performance in biomedical and forensic datasets [65, 67, 68]. Feature importance was computed using three tree-based ensemble approaches (Random Forest, Extra Trees, and XGBoost), where variables were ranked according to mean impurity reduction scores to determine the optimal features for sex estimation using various machine learning models. Variables consistently ranked within the top five positions across methods were selected for subsequent model development.

## Model development

**Fig. 2:**
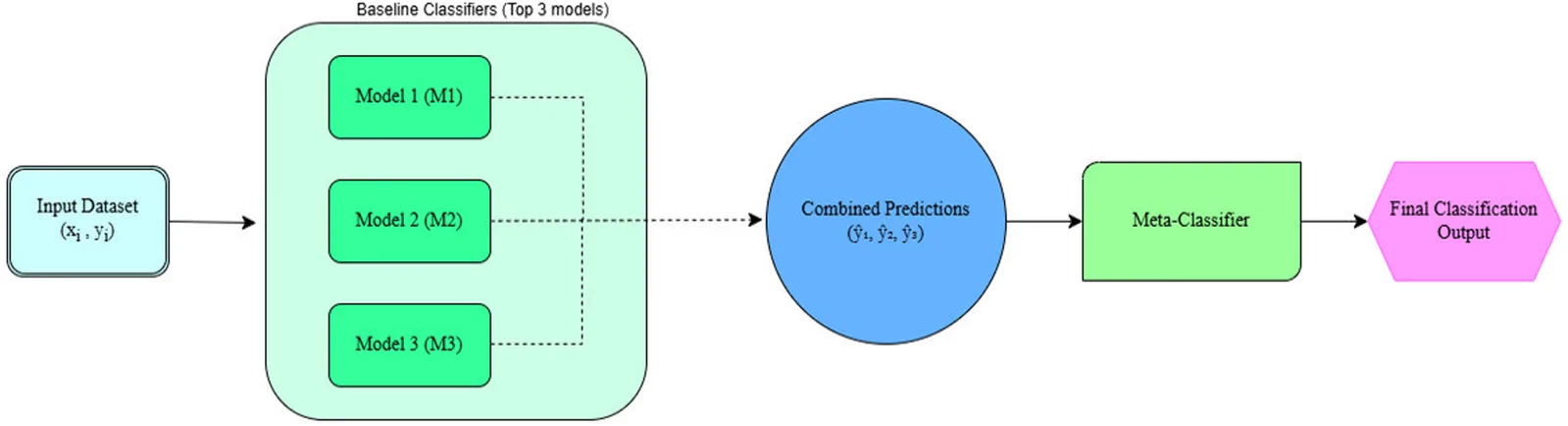
Stacking model architecture

Several machine learning algorithms were evaluated for sex classification, including Gradient Boosting, XGBoost, Extra Trees, K-Nearest Neighbors (KNN), AdaBoost, Random Forest, Linear Discriminant Analysis (LDA), and Logistic Regression Analysis (LRA). These models were trained using the top-ranked feature sets. For the stacking framework, the three highest-performing base learners (Extra Trees, Random Forest, and XGBoost) were selected. Their prediction probabilities served as input features to the meta-learning stage. Logistic Regression was identified as the optimal meta-classifier after evaluating seven candidate algorithms and was therefore used as the final classifier. This process is visualized in Fig. 2, illustrating how input features and model outputs are combined in the stacking framework.

A five-fold cross-validation strategy was used to create training data for the meta-classifier. In each round, four folds trained the base models, and the fifth was used for validation. Each base model generated a probability distribution for the target classes, producing a set of predicted probabilities for each input from the ensemble of models (M).

2 $$\:\begin{array}{c}\begin{array}{c}{\:P}^M\left(x\right)\end{array}\\=\left(P^M\left(x\right),P^M\left(x\right),\dots\:\dots\:.,P^M\left(x\right)\right)\end{array}\\\\\\$$

**Fig. 3:**
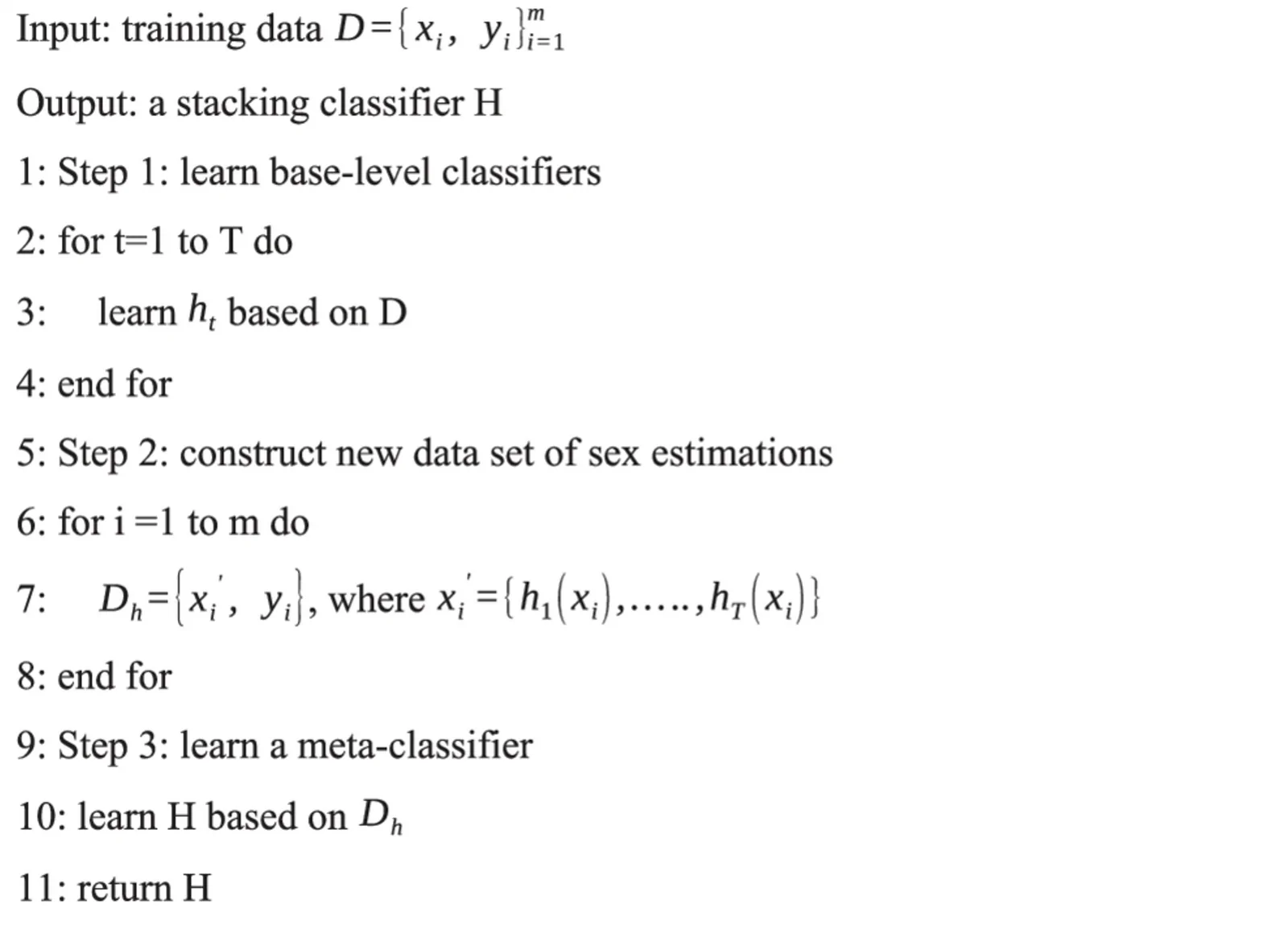
Stacking classification

Here, $\:{(c}_{1},{c}_{2},\dots\:\dots\:,{c}_{n})\:$represents the set of all possible class labels, and $\:{P}^{M}\left(x\right)$ refers to the probability that instance x belongs to class $\:{c}_{j}$, as estimated by classifier M (see Eq. 2). The class $\:{c}_{i}$ with the highest predicted probability $\:{P}^{{M}_{j}}\left(x\right)$ is selected as the output of classifier M. For the meta-classifier $\:{M}_{f},$ the input features consist of the class probabilities predicted by each base-level model. Specifically, the inputs are the values $\:{P}^{{M}_{j}}\left(x\right)$, i = 1,., n (for all classes) and j = 1,., p. (for all base classifiers). The stacking procedure is outlined in Fig. 3.

## Performance metrics

To assess model performance in classifying sex, various classifiers were tested using top-ranked features from the test set. The best model was evaluated across different feature combinations by calculating the receiver operating characteristic (ROC) area under the curve (AUC) and standard metrics, including Accuracy, Precision, Sensitivity (Recall), Specificity, and F1-Score, as defined in Eqs. (2–6). A five-fold cross-validation was applied, with each fold using 80% of the data for training and 20% for testing, repeated five times to ensure full dataset coverage. Performance metrics were calculated as weighted averages with 95% confidence intervals, based on confusion matrices combining results from all folds. In this analysis, True Positives (TP) represent correctly predicted males, True Negatives (TN) correctly predicted females, False Negatives (FN) are males misclassified as females, and False Positives (FP) are females misclassified as males.

3 $$\:Accuracy=\frac{TP+TN}{TP+TN+FP+FN}$$

4 $$\:Precision=\frac{TP}{TP+FP}\:\:\:\:\:$$

5 $$\:Sensitivity=\frac{TP}{TP+FN}$$

6 $$\:Specificity=\frac{TN}{TN+FP}\:\:\:\:$$

7 $$\:F1-score=2\frac{Precision\times\:Sensitivity}{Precision+Sensitivity}$$

### Model explainability

Interpreting how a model makes sex estimations is essential, especially when those decisions have real-world consequences. While high-performing models like ensemble methods and deep neural networks handle complex data well, they are often criticized for being opaque or “black boxes.” This raises a common challenge: balancing accuracy with interpretability. Although several methods have been developed to improve model transparency, selecting the right one remains complex. One of the most widely used interpretability tools is SHAP (SHapley Additive exPlanations). Grounded in game theory, SHAP explains sex‑estimation model outputs by assigning Shapley values to each input feature, indicating its contribution to an individual estimate. This approach allows for consistent, fair, and detailed insights into how features influence outcomes, even in complex, non-linear models.

## Results

In the cranial dataset, complete measurements were available for all eight variables in both male and female groups, with each group comprising 200 individuals (*n* = 200 per sex). A consistent pattern emerged in which males exhibited higher mean values than females across most cranial dimensions (Table 4). For example, the mean for Cra_01 was 189.78 mm in males compared to 184.41 mm in females, and for Cra_05, males averaged 136.25 mm while females averaged 131.23 mm. These differences highlight clear sexual dimorphism in cranial morphology.

The pelvic bone dataset included measurements from 140 male and 140 female individuals (*n* = 140 per group), also showing complete data for all eleven pelvic variables (Table 5). Similar to the cranial data, males consistently displayed greater mean values across all pelvic dimensions, reinforcing the presence of sexual dimorphism in pelvic bone morphology. These observed differences in both cranial and pelvic metrics support the utility of osteometric variables for sex estimation.

**Table 4:** Descriptive statistics of cranial measurements^58^

Sex	Statistic	Cra_01	Cra_04	Cra_05	Cra_06	Cra_11	Cra_12	Cra_14	Cra_15
Female	n	200	200	200	200	200	200	200	200
	Max (mm)	203.5	149.3	144.8	113.5	82.5	131.6	55.5	36.0
	Mean (mm)	184.4	128.2	131.2	95.6	67.5	116.5	47.6	28.1
	Median (mm)	183.8	127.8	130.8	96.0	67.5	116.1	47.6	28.1
	Minimum (mm)	170.4	114.6	119.9	85.2	56.5	105.9	27.5	21.5
	Q1 (mm)	180.7	124.4	128.3	93.7	64.4	113.5	45.4	26.5
	Q3 (mm)	187.9	131.1	134.3	97.6	70.5	119.0	50.0	29.4
	Std Dev	5.9	5.3	4.7	4.3	4.3	4.3	3.4	2.4
Male	n	200	200	200	200	200	200	200	200
	Max (mm)	206.8	154.4	153.1	113.9	85.4	133.9	60.4	37.2
	Mean (mm)	189.8	135.3	136.3	100.2	71.4	120.5	50.8	28.8
	Median (mm)	190.0	135.5	136.5	100.1	71.5	120.6	50.6	28.8
	Minimum (mm)	165.5	120.2	121.1	88.5	58.2	101.8	42.8	22.2
	Q1 (mm)	185.5	131.8	132.3	96.8	68.4	117.5	48.7	27.1
	Q3 (mm)	194.3	138.6	140.0	103.8	74.2	124.3	52.6	30.4
	Std Dev	7.3	5.6	6.0	5.0	4.5	5.6	3.1	2.6

**Table 5:** Descriptive statistics of pelvic bone measurements [59]

Sex	Statistic	AceDia	GSNb	GSNd	IlB	IsL	OFH	OFW	PuH	PuL	PuW	THox
Female	n	140	140	140	140	140	140	140	140	140	140	140
	Max (mm)	59.3	66.5	38.6	180.8	124.6	47.7	58.6	40.4	119.0	33.8	233.5
	Mean (mm)	48.4	52.6	31.0	149.4	102.3	34.0	49.2	32.2	98.8	24.5	195.2
	Median (mm)	48.5	53.2	30.8	149.6	101.7	33.7	49.3	32.3	98.8	24.5	194.3
	Min (mm)	40.8	38.9	22.8	124.0	87.7	20.9	30.5	24.6	79.1	13.6	166.9
	Q1 (mm)	45.9	48.3	28.6	143.8	98.1	31.8	46.4	29.5	94.0	22.1	187.6
	Q3 (mm)	50.2	56.5	33.7	155.3	106.7	36.4	52.0	34.6	104.4	26.7	201.3
	Std Dev	3.4	5.0	3.5	9.5	6.7	3.7	4.6	3.7	7.3	3.3	11.4
Male	n	140	140	140	140	140	140	140	140	140	140	140
	Max (mm)	60.5	59.5	43.8	179.0	144.0	51.5	61.8	47.3	110.3	36.2	237.0
	Mean (mm)	53.7	47.3	31.0	153.1	109.8	34.6	51.0	34.7	94.9	21.4	208.0
	Min (mm)	44.7	35.8	20.4	131.5	91.3	27.3	31.0	26.0	74.4	16.8	181.9
	Q1 (mm)	51.9	44.2	28.1	146.9	104.3	31.6	48.1	32.2	89.9	19.6	200.1
	Q3 (mm)	55.7	50.8	33.0	159.4	114.0	36.7	53.7	37.4	100.6	22.9	216.1
	Std Dev	3.0	4.6	4.0	9.7	7.7	3.7	4.7	3.8	8.0	2.9	11.4

## Machine learning analysis

### Best feature combination for sex estimation using cranial measurements

**Fig. 4:**
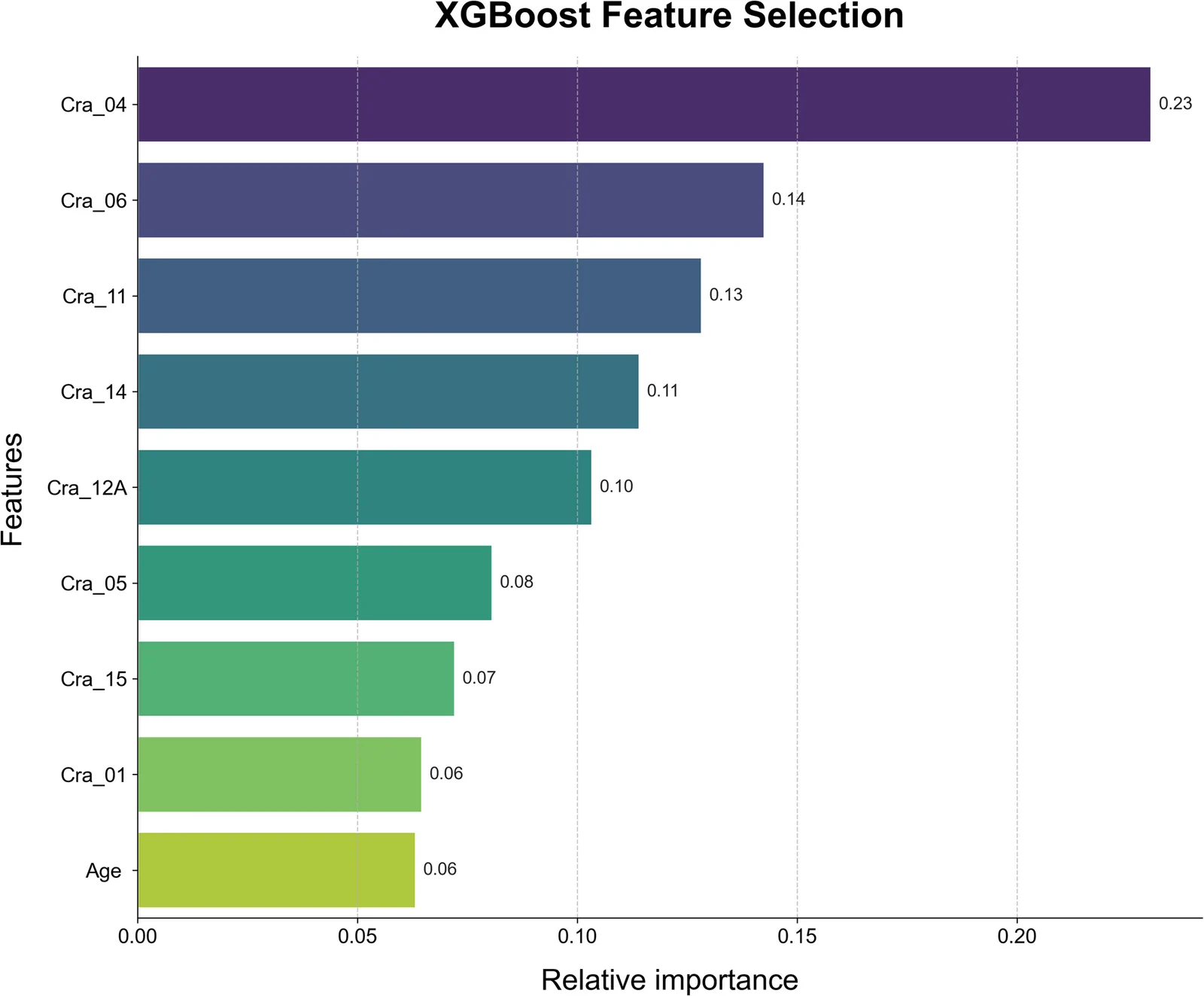
Feature importance bar plot for cranial measurements

As illustrated in Fig. 4, feature selection performed on the cranial dataset identified five variables: Cra_04, Cra_06, Cra_11, Cra_14, and Cra_12 as the most effective for distinguishing between male and female individuals. These selected features represent critical variables of cranial morphology that exhibit strong sexual dimorphism. They were subsequently used as input variables for training and evaluating multiple machine learning classifiers aimed at sex estimation.

As shown in Table 6, the Extra Trees classifier emerged as the best-performing individual (base) learner, achieving an accuracy of 86.75%. It also recorded equal values for precision, recall (sensitivity), specificity, and F1-score (each at 86.75%), indicating balanced and reliable performance across evaluation metrics.

However, overall classification performance was significantly improved through the use of a stacking ensemble approach. This method combined the sex estimations of the top-performing base models to train a meta-classifier, resulting in superior predictive accuracy. The stacked model achieved an accuracy of 94.25%, a precision of 94.28%, and an F1-score of 94.24%, thereby outperforming all individual classifiers. These findings highlight that the selected cranial features, when integrated into a stacking ensemble framework, provide a robust and highly accurate solution for sex classification, demonstrating the added value of ensemble learning in forensic anthropology.

**Table 6:** Performance comparison of various classifiers and meta-classifiers for sex estimation using cranial and pelvic measurements

Dataset	Metrics	Random Forest	KNN	Cat Boost	XGB	Logistic Regression	LDA	Extra Trees	Meta-classifier
Cranial	Accuracy	80.25	80.25	80.25	80.00	82.75	83.00	86.75	**94.25**
	Precision	80.25	80.32	80.27	80.10	82.75	83.00	86.75	**94.28**
	Recall	80.25	80.25	80.25	80.00	82.75	83.00	86.75	**94.25**
	Specificity	80.25	80.25	80.25	80.00	82.75	83.00	86.75	**94.25**
	F1-Score	80.25	80.24	80.24	79.98	82.74	83.00	86.75	**94.24**
Pelves	Accuracy	86.07	86.43	86.07	82.86	90.36	90.36	93.21	**96.07**
	Precision	86.08	86.70	86.16	82.88	90.36	90.38	93.32	**96.09**
	Recall	86.07	86.42	86.07	82.86	90.36	90.36	93.21	**96.07**
	Specificity	86.07	86.42	86.07	82.86	90.36	90.36	93.21	**96.07**
	F1-Score	86.07	86.40	86.06	82.86	90.36	90.36	93.21	**96.07**

### Best feature combination for sex estimation using pelvic bone measurements

**Fig. 5:**
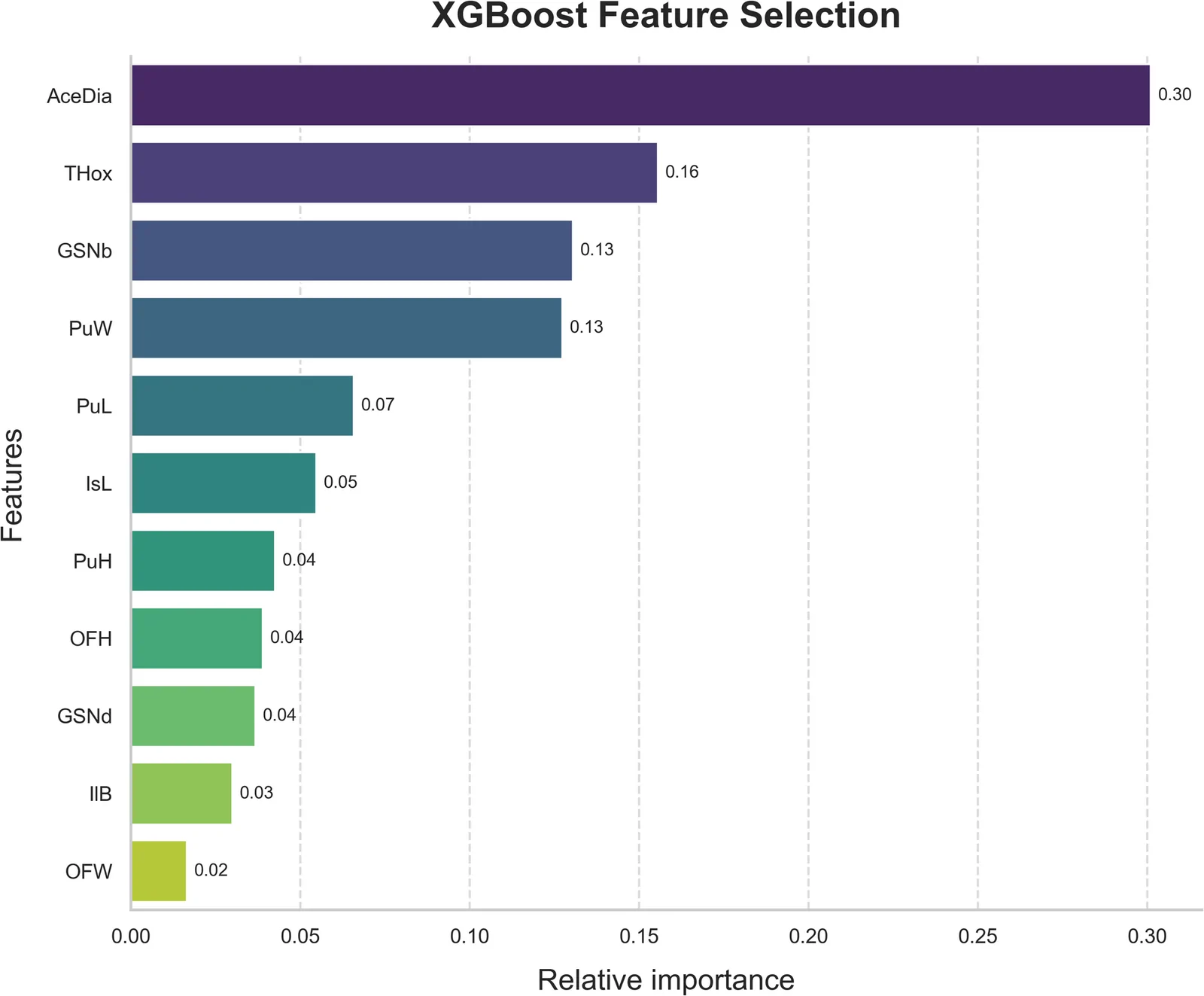
Feature importance bar plot for pelvic measurements

In the pelvic bone dataset, the best performing features identified through feature selection were AceDia, THox, GSNb, and PuW (Fig. 5). These variables demonstrated strong sexual dimorphism and were particularly effective for distinguishing between sexes. As indicated in Table 6, the Extra Trees classifier once again emerged as the best-performing base model, achieving 93.21% across all key performance metrics: accuracy, precision, recall, and F1-score.

Building on this, the stacking ensemble model further enhanced classification performance. By integrating sex estimations from the top base learners, the meta-classifier achieved an accuracy of 96.07%, with a precision of 96.09% and an F1-score of 96.07%, surpassing the results of any individual model. This confirms that the selected pelvic bone variables, when combined with an ensemble learning approach, provide a highly accurate method for sex estimation.

### Development and validation of different ML and stacking models

To perform sex classification using cranial and pelvic measurements, a variety of classical machine learning (ML) algorithms were developed and rigorously evaluated. The goal was to compare the predictive performance of individual models and determine whether a stacking ensemble approach could further improve classification accuracy. Seven widely used supervised ML algorithms were implemented: Logistic Regression (LR), Support Vector Machine (SVM), K-Nearest Neighbors (KNN), Random Forest (RF), Extra Trees (ET), and XGBoost (XGB). Each algorithm was trained separately on the cranial and pelvic datasets, both of which were pre-processed using Z-score normalization to standardize feature scales.

To ensure fair and robust evaluation, stratified 5-fold cross-validation was applied. This technique maintained an equal representation of males and females in each fold, minimizing potential bias and enhancing the generalizability of the models. Hyperparameters for each classifier were optimized through grid search to maximize performance. Model effectiveness was measured using key evaluation metrics: accuracy, precision, recall (sensitivity), F1-score, and specificity.

Beyond individual classifiers, a stacking ensemble model was constructed to leverage the strengths of multiple learners. This method involved training a group of top-performing base models and combining their output probabilities as input features for a meta-classifier. The meta-classifier then generated the final sex estimation. This ensemble strategy was designed to reduce overfitting, enhance model stability, and improve generalization to new, unseen data.

The stacking ensemble consistently outperformed the individual classifiers in terms of predictive performance. For the cranial dataset, the Extra Trees classifier achieved an accuracy of 86.75%, while the stacking model improved this to 94.25%, along with notable gains in precision, recall, and F1-score. A similar pattern was observed in the pelvic dataset, where the Extra Trees model reached an accuracy of 93.21%, and the stacking ensemble further increased it to 96.07%. These results, detailed in Table 6, underscore the effectiveness of the stacking approach. By integrating the strengths of multiple classifiers, the ensemble model captures diverse decision patterns, leading to more accurate and reliable sex classification outcomes.

**Fig. 6:**
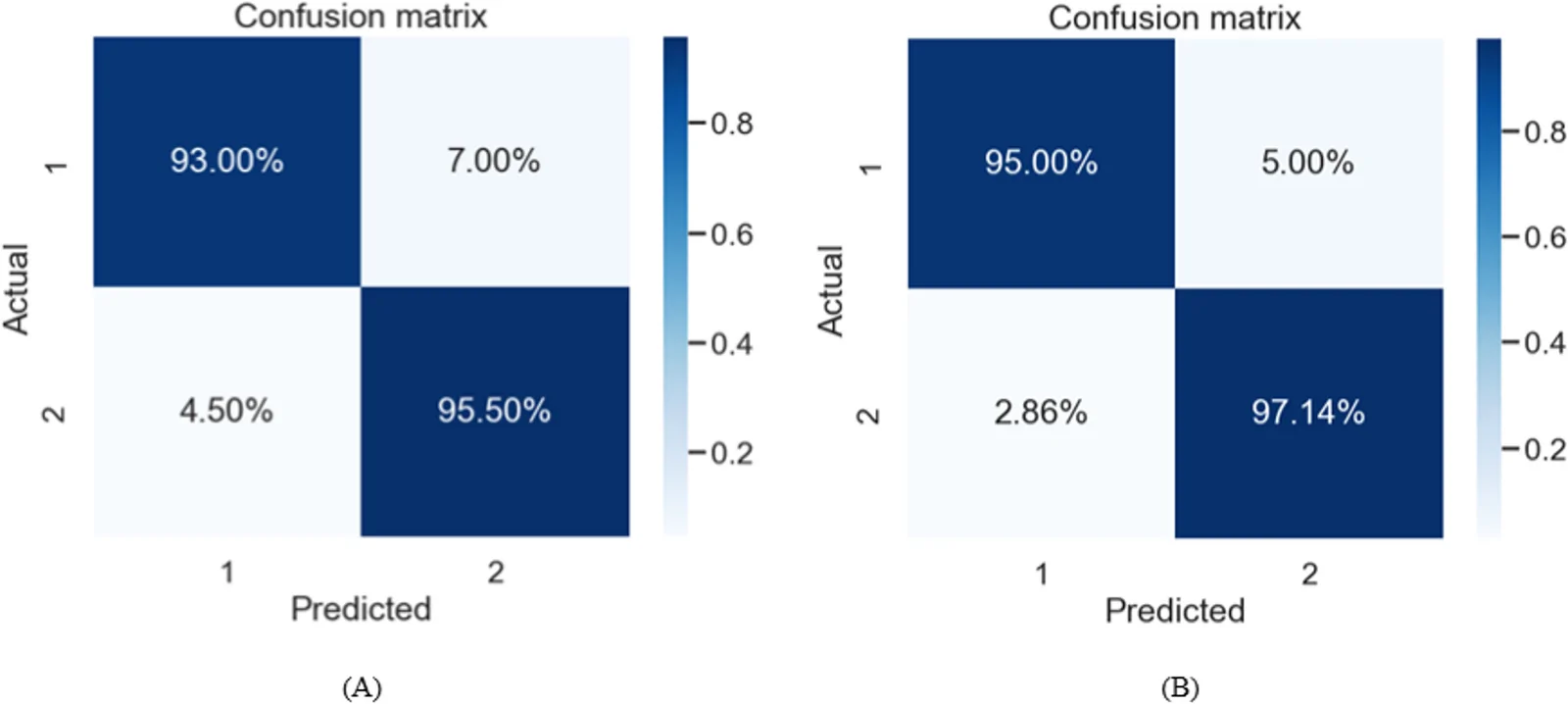
Confusion matrix for sex estimation model using (**A**) cranial measurements and (**B**) pelvic measurements

Figure 6(A) presents the confusion matrix for the sex classification model based on cranial measurements, while Fig. 6(B) illustrates the matrix for the model using pelvic measurements. The cranial model accurately identified 93.00% of female cases and 95.50% of male cases, with corresponding misclassification rates of 7.00% and 4.50%. In comparison, the pelvic model demonstrated superior performance, correctly classifying 95.00% of females and 97.14% of males, with lower misclassification rates of 5.00% and 2.86%, respectively.

**Fig. 7:**
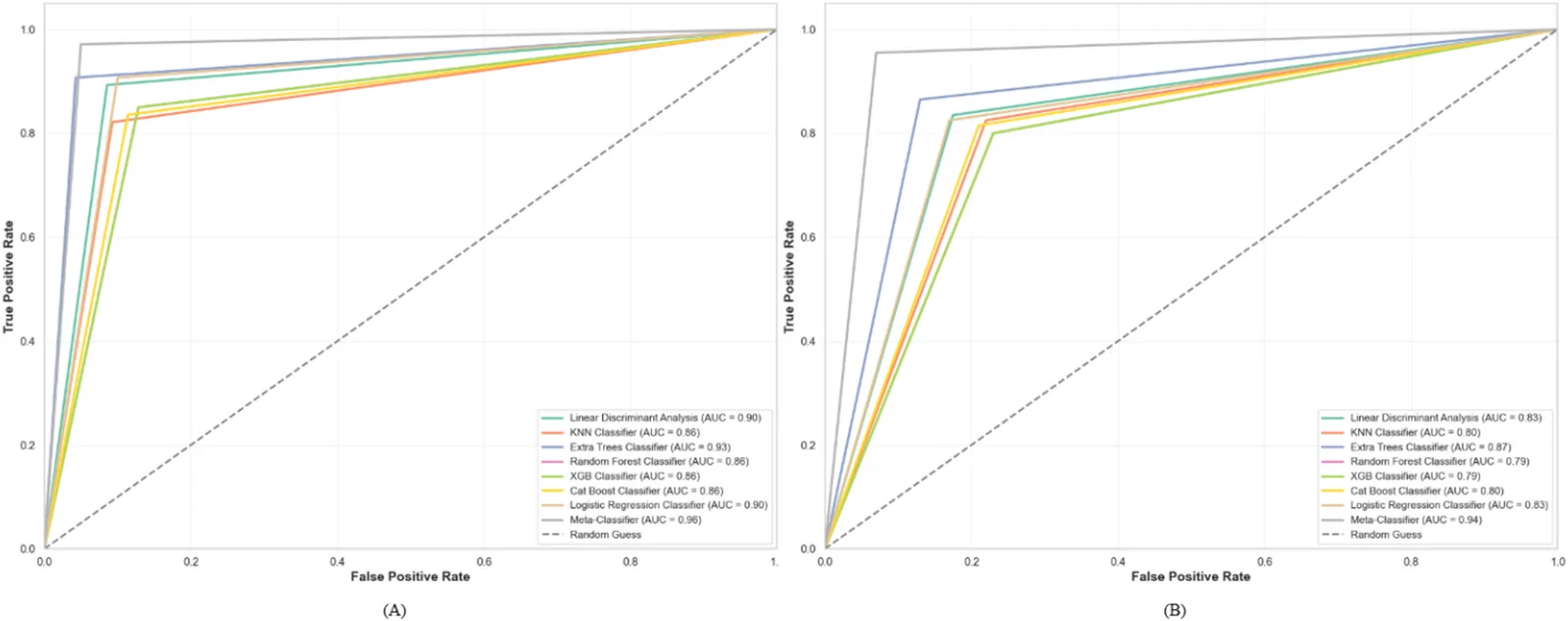
ROC curve for sex estimation classifier using different using (**A**) cranial measurements and (**B**) pelvic measurements

Figure 7A and B illustrate the ROC curves and corresponding Area Under the Curve (AUC) values for sex classification across different machine learning models. In Fig. 7(A), which represents models trained on cranial measurements, the Meta-Classifier achieved the highest AUC of 0.96. This was followed by the Extra Trees classifier with an AUC of 0.93, while both Linear Discriminant Analysis (LDA) and Logistic Regression recorded AUC values of 0.90. Figure 7(B) shows the results for pelvic measurements, where the Meta-Classifier again led with an AUC of 0.94, outperforming Extra Trees (0.87) and LDA/Logistic Regression (0.83).

### Model explainability

**Fig. 8:**
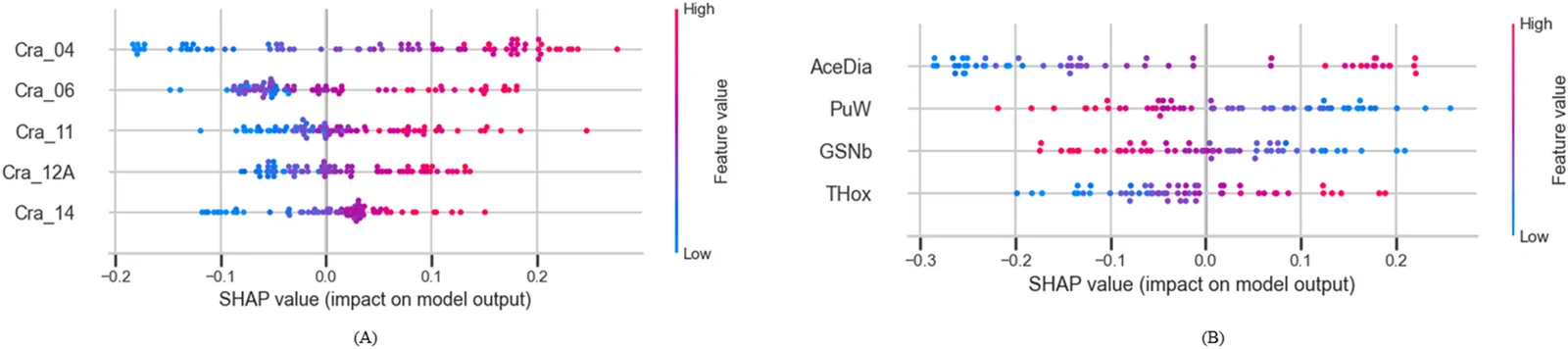
SHAP summary plot for sex estimation model using (**A**) cranial measurements and (**B**) pelvic measurements

Figures 8(A) and 7(B) display SHAP summary plots for the cranial and pelvic datasets, respectively, illustrating the contribution of each feature to the model’s sex classification estimations. In Fig. 8(A), cranial variables such as Cra_04, Cra_06, and Cra_11 exhibit consistently high SHAP values, indicating their strong importance in influencing model output. For example, higher Cra_04 values tend to drive sex estimations toward one sex, while lower values shift sex estimation toward the other, a pattern similarly observed for Cra_06 and Cra_11. Additional features like Cra_14 and Cra_12 also play a role, although their SHAP value ranges are narrower, suggesting a moderate but still meaningful impact on the model’s decisions.

In Fig. 8(B), the SHAP values for pelvic measurements reveal even clearer distinctions in predictive influence. AceDia and THox stand out with the largest SHAP spreads, signifying a strong effect on classification outcomes. GSNb and PuW also contribute significantly, with SHAP plots revealing complex, non-linear effects, where both low and high values influence the model differently. These findings highlight SHAP’s utility in uncovering how individual features, even in non-linear models, drive predictive behaviour.

## Discussion

This study provides an in‑depth examination of classical machine‑learning (ML) algorithms for sex estimation using CT‑derived cranial and pelvic measurements in a contemporary South African population. The findings reaffirm that both skeletal regions display strong sexual dimorphism in the cranial and pelvic bone morphology, corroborating previous osteometric and morphometric studies [2, 5, 18, 30, 37]. Unlike earlier studies that predominantly relied on classical statistical approaches such as discriminant function and logistic regression analyses ²^,^¹⁸, which, while reliable, are limited by assumptions of normality and linearity, this study employs ML techniques to better model complex, non-linear relationships. However, it is important to note that, like traditional methods, ML models may still be influenced by population-specific characteristics and may not generalise equally well across different populations.

ML models provide greater flexibility by capturing complex, non-linear relationships between skeletal measurements and sex classification outcomes. The application of ML represents a methodological advancement in forensic anthropology, especially in cases where traditional models may fall short. Consistent with previous studies, this research confirms the marked sexual dimorphism in cranial and pelvic bone morphology among individuals of Black South Africans. Importantly, ensemble-based ML models, particularly stacking techniques, delivered significantly higher predictive accuracy compared to both individual classifiers and traditional statistical models.

The stacking ensemble model, which integrates sex estimations from the top three base classifiers, Extra Trees, XGBoost, and Random Forest, achieved the highest classification accuracies: 94.25% for cranial data and 96.07% for pelvic data. These results exceeded those of any standalone model, including Extra Trees, which was the strongest individual performer. This highlights the power of ensemble learning, which combines multiple algorithms to enhance generalizability, reduce model bias and variance, and improve overall robustness in biological classification tasks [43–46],

Pelvic measurements outperformed cranial measurements in all classification metrics, echoing prior research that consistently identifies the pelvis as the most sexually dimorphic skeletal region [2, 18, 37, 54, 55]. Among the pelvic features, AceDia, THox, GSNb, and PuW were identified as the most informative, with consistently high feature importance scores and SHAP values. For cranial features, Cra_04, Cra_06, Cra_11, Cra_14, and Cra_12 were the most predictive. These variables correspond to well-documented sexually dimorphic traits in anthropological literature [2, 8–12].

The use of SHAP for model explainability added a critical interpretive layer to the analysis. SHAP values quantified the contribution of individual features to model sex estimations and effectively addressed the “black box” criticism often associated with complex ML algorithms. This interpretability is particularly relevant in forensic contexts, where transparent decision-making is essential for legal admissibility and expert testimony [46, 47].

Compared to traditional statistical approaches, the ML models developed in this study offer several advantages. First, ML algorithms are non-parametric and do not require assumptions about the distribution or linearity of the data, limitations that commonly affect logistic regression and discriminant analysis [18]. Second, the stacking ensemble classifier demonstrated high performance metrics (accuracy, precision, recall, specificity) despite the presence of multicollinearity and nonlinear relationships in the data. Third, the incorporation of feature selection via tree-based algorithms (Random Forest and Extra Trees), along with hyper parameter tuning through grid search and cross-validation, contributed to the accuracy, reliability, and reproducibility of the models [45, 47].

Nonetheless, this study has limitations. The dataset was restricted to high-resolution CT scans of contemporary Black South Africans, which limits the generalizability of the models to other populations. Sexual dimorphism varies significantly across ancestral groups, shaped by genetic, environmental, and cultural influences [5]. Therefore, the equations and models derived here should be cautiously applied outside the demographic context in which they were developed. Furthermore, while CT imaging ensures precision and reproducibility, it may not be widely available in all forensic or archaeological settings, especially in resource-limited environments [37].

For practical forensic implementation, the developed ML models may be used as decision-support tools rather than stand-alone decision systems [46]. The final output generated by the stacking classifier provides prediction probabilities for male and female classifications. In forensic casework, predictions associated with high confidence levels (e.g., ≥ 0.90 probability) may be considered strong support for sex estimation, whereas predictions close to the decision threshold (e.g., probabilities between 0.40 and 0.60) should be interpreted cautiously as uncertain classifications [69–71]. Such uncertain cases should be evaluated in conjunction with additional biological profile indicators, including age estimation, stature reconstruction, dental records, and morphological assessments. Misclassification risks should also be interpreted within the medico-legal context, recognizing that false-positive and false-negative predictions may have different practical consequences depending on investigative circumstances. Therefore, these models are intended to assist expert interpretation rather than replace anthropological expertise.

Although stratified five-fold cross-validation was used to reduce overfitting and improve internal reliability [71, 72], the present study did not include independent external validation using unseen datasets. Consequently, the reported performance metrics may overestimate model performance under real-world conditions. External validation using independent skeletal datasets from different geographic regions, imaging systems, and population groups will be essential before these models can be adopted for routine forensic implementation. Testing the models on dry skeletal remains and lower-resolution imaging modalities (e.g., radiographs or 3D surface scans) would broaden their practical utility. Moreover, the integration of geometric morphometric data with ML pipelines, already being explored in emerging studies [6–8]. Holds promise for improving model generalizability, morphological coverage, and robustness in partial remains.

## Conclusions

This study demonstrates that ML models, particularly stacking ensemble classifiers, can substantially enhance the accuracy and reliability of sex estimation from cranial and pelvic skeletal measurements. These models outperform traditional statistical approaches and provide an interpretable, scalable, and reproducible tool for forensic anthropology and bioarchaeological investigations.

## Data Availability

No datasets were generated or analysed during the current study.
